# DPP8 Selective Inhibitor Tominostat as a Novel and Broad-Spectrum Anticancer Agent against Hematological Malignancies

**DOI:** 10.3390/cells12071100

**Published:** 2023-04-06

**Authors:** Shohei Kikuchi, Akinori Wada, Yusuke Kamihara, Kosuke Okazaki, Paras Jawaid, Mati Ur Rehman, Eiji Kobayashi, Takeshi Susukida, Tomoki Minemura, Yoshimi Nabe, Noriaki Iwao, Tatsuhiko Ozawa, Ryo Hatano, Mitsugu Yamada, Hiroyuki Kishi, Yuji Matsuya, Mineyuki Mizuguchi, Yoshihiro Hayakawa, Nam H. Dang, Yasumitsu Sakamoto, Chikao Morimoto, Tsutomu Sato

**Affiliations:** 1 Department of Hematology, Faculty of Medicine, Academic Assembly, University of Toyama, 2630 Sugitani, Toyama 930-0194, Japan; 2 Center for Clinical Research, Toyama University Hospital, 2630 Sugitani, Toyama 930-0194, Japan; 3 Department of Biological and Biomedical Sciences, The Aga Khan University, Karachi 74800, Pakistan; 4 Department of Immunology, Faculty of Medicine, Academic Assembly, University of Toyama, 2630 Sugitani, Toyama 930-0194, Japan; 5 Section of Host Defences, Institute of Natural Medicine, University of Toyama, 2630 Sugitani, Toyama 930-0194, Japan; 6 Department of Hematology, Juntendo University Shizuoka Hospital, 1129 Nagaoka, Izunokuni City, Shizuoka 410-2295, Japan; 7 Department of Therapy Development and Innovation for Immune Disorders and Cancers, Graduate School of Medicine, Juntendo University, 2-1-1 Hongo Bunkyo-ku, Tokyo 113-8421, Japan; 8 JEM Utilization Center Human Spaceflight Technology Directorate, Japan Aerospace Exploration Agency (JAXA), 2-1-1 Sengen, Tsukuba-shi 305-8505, Japan; 9 Faculty of Pharmaceutical Sciences, University of Toyama, 2630 Sugitani, Toyama 930-0194, Japan; 10 Division of Hematology/Oncology, University of Florida, Gainesville, FL 32610, USA; 11 School of Pharmacy, Iwate Medical University, 1-1-1 Idaidori, Yahaba 028-3694, Japan

**Keywords:** DPP8, inhibitor, anticancer agent, hematological, malignancies

## Abstract

DPP8/9 inhibition induces either pyroptotic or apoptotic cell death in hematological malignancies. We previously reported that treatment with the DPP8/9 inhibitor 1G244 resulted in apoptotic cell death in myeloma, and our current study further evaluates the mechanism of action of 1G244 in different blood cancer cell lines. Specifically, 1G244 inhibited DPP9 to induce GSDMD-mediated-pyroptosis at low concentrations and inhibited DPP8 to cause caspase-3-mediated-apoptosis at high concentrations. HCK expression is necessary to induce susceptibility to pyroptosis but does not participate in the induction of apoptosis. To further characterize this DPP8-dependent broad-spectrum apoptosis induction effect, we evaluated the potential antineoplastic role for an analog of 1G244 with higher DPP8 selectivity, tominostat (also known as 12 m). In vitro studies demonstrated that the cytotoxic effect of 1G244 at high concentrations was enhanced in tominostat. Meanwhile, in vivo work showed tominostat exhibited antitumor activity that was more effective on a cell line sensitive to 1G244, and at higher doses, it was also effective on a cell line resistant to 1G244. Importantly, the weight loss morbidity associated with increasing doses of 1G244 was not observed with tominostat. These results suggest the possible development of novel drugs with antineoplastic activity against selected hematological malignancies by refining and increasing the DPP8 selectivity of tominostat.

## 1. Introduction

The S9b serine protease family has the ability to cleave Xaa-Pro dipeptides from N-termini of their substrates, and these enzymatic members include dipeptidyl peptidase-4 (DPP4), DPP8, DPP9, and fibroblast activation protein (FAP) [1].

For example, DPP4 cleaves the pancreatic polypeptide family, including neuropeptide Y and peptide YY, several members of the glucagon family, and certain chemokines as natural substrates [2,3]. Of these substrates, insulino-tropic hormone (incretin) glucagon-like peptide 1 (GLP-1) is of particular interest, which is cleaved and inactivated by DPP4 to antagonize insulin secretion. Small-molecule inhibitors of DPP4 have been developed as well-established therapies for the treatment of type II diabetes [4]. DPPs are therefore an important class of enzymes that are potential therapeutic targets for human diseases due to their highly selective and limited proteolytic activities.

Several proteins have been identified as substrates of DPP8 and DPP9 [1,5,6,7,8,9,10]. DPP8 substrates include inflammatory protein-10 (IP10), interfering T-cell chemokines (ITAC), and chemokines stromal cell-derived factor (SDF-1) [10], while DPP9 cleaves C-X-C motif chemokine 10 (CXCL10/IP10), S100-A10, SET, nucleobindin-1 (NUCB1), and interleukin-1 receptor antagonist protein (IL-1RA) as substrates [8]. In addition, numerous studies have shown that DPP8/9 is involved in immune system regulation and inflammation by the cleavage of these substrates [11].

DPP8 and DPP9 localize intracellularly in contrast to DPP4, which has an extracellular catalytic domain. With significant homology among these enzymes (79% amino acid similarity and 61% amino acid identity) [12], no small-molecule compounds have heretofore been identified to selectively inhibit only one of these proteins. On the other hand, inhibitors which simultaneously inhibit both DPP8 and DPP9 have been demonstrated to be potential therapeutic agents for selected hematologic malignancies, as described below.

The DPP family nonselective inhibitor Val-boroPro (talabostat) triggers a lytic form of programmed cell death known as pyroptosis in human acute myeloid leukemia (AML) cell lines and primary AML samples, an effect that is dependent on DPP9 inhibition [13]. The inhibition of DPP9 in mouse monocytes and macrophages by talabostat causes the autoproteolysis of the function-to-find (FIIND) domain in the inflammasome sensor protein, nucleotide-binding domain, and leucine-rich repeat pyrin containing 1b (Nlrp1b) and the cleavage of gesdermin D (GSDMD) by caspase-1 activated by this autoproteolysis induces pyroptosis [14]. Interestingly, DPP9 functions as an endogenous inhibitor of Nlrp1b rather than as a protease in this series of reactions [15]. In human AML cells, NLRP1 and caspase recruitment domain family member 8 (CARD8) function as human homologs of mouse Nlrp1b, and the expression levels of caspase-1 and CARD8 determine the sensitivity to talabostat [13].

Alternatively, 1G244 is a specific inhibitor of DPP8 and DPP9 [11]. We have reported that 1G244 triggers caspase-3-activation-mediated apoptosis in multiple myeloma (MM) cell lines and primary MM samples, and that this effect may be dependent on DPP8 inhibition [16]. However, cell death induced by 1G244 in the AML cell line THP-1 is not dependent on DPP8/9 inhibition but is the result of an unidentified off-target effect [14]. In view of these contradictory findings, we conducted the studies described in this paper to further investigate 1G244-induced cell death.

On the other hand, 12 m, a 1G244 methylpiperazine analog, has been reported to be highly selective for DPP8 [17]. Based on our hypothesis that the antitumor effect of 1G244 depends on DPP8 inhibition, we would expect 12 m to exhibit potent antineoplastic activity. For our current work, we have named 12 m as tominostat and have investigated its potential role as an anticancer drug.

In this study, we found that high concentrations of 1G244 induce caspase-3-activation-mediated apoptosis that is dependent on DPP8 inhibition. We also demonstrated that the higher level of selectivity for DPP8 by tominostat enhances its apoptotic effect and that it may be a more potent anticancer drug with an improved in vivo weight loss toxicity profile.

## 2. Materials and Methods

### 2.1. Cell Culture

T cell non-Hodgkin’s lymphoma cell line KARPAS299 was supplied by the European Collection of Authenticated Cell Cultures (ECACC). Multiple myeloma cell lines, MM.1S and RPMI8226; Burkitt’s lymphoma cell lines, Daudi, Raji, and NAMALWA; the acute myelogenous leukemia cell line, KG1; the acute T cell leukemia cell line, Jurkat; and the chronic myelogenous leukemia cell line, K562, were supplied by American Type Culture Collection (ATCC). Acute monocytic leukemia cell lines, THP-1, MOLM-13, and NOMO-1; and the myelodysplastic syndrome cell line, SKM-1, were supplied by Japanese Collection of Research Bioresources Cell Bank (JCRB). All these cell lines were maintained in RPMI 1640 (Gibco BRL) supplemented with 10% heat-inactivated fetal bovine serum (Sigma), 100 µg/mL streptomycin, and 100 U/mL penicillin.

### 2.2. Lentivirus and Transduction

The shRNA lentivirus, MISSION Lentiviral Transduction Particles, SHCLNV was purchased from Sigma-Aldrich. The lentivirus vector system is composed of the vector pLKO.1-Puro-CMV-tGFP. The control is MISSION pLKO.1-Puro-CMV-tGFP Positive Control Transduction Particles, SHC003V. DPP8 KD1, TRCN0000300778; DPP8 KD2, TRCN0000300777. DPP9 KD1, TRCN0000075265; DPP9 KD2, TRCN0000075264. HCK KD1, TRCN0000320535: HCK KD2, TRCN0000381826. The human HCK gene overexpressing lentivirus, HCK (NM_002110) Human Tagged ORF Clone Lentiviral Particle, RC217022L4V was purchased from OriGene. The lentivirus vector system is composed of the vector pLenti-C-mGFP-P2A-Puro. The control is Lenti ORF control particles of pLenti-C-mGFP-P2A-Puro, PS100093V. Lentivirus transduction and stable cell line selection were performed according to the manufacturer’s instructions.

### 2.3. Reagents

Puromycin and 1G244 were purchased from Sigma-Aldrich. Talabostat, Disulfiram, and Necrostatin-1 were purchased from MedChemexpress. Necrosulfonamide was purchased from Santa Cruz Biotechnology. Z-DEVD-FMK and Z-VAD-FMK were purchased from Medical & Biological Laboratories. Tominostat (12 m) was prepared according to previously published work [17].

### 2.4. Cellular Cytotoxicity

The viable cell number was quantified using a Premix WST-1 Cell Proliferation Assay System (TaKaRa, Shiga, Japan) according to the manufacturer’s instructions. The level of cytotoxicity was also quantified by measuring the level of lactate dehydrogenase (LDH) released from damaged cells using a Cytotoxicity LDH Assay Kit-WST (Dojindo, Kumamoto, Japan) according to the manufacturer’s instructions.

### 2.5. Western Blot Analyses

Cells were lysed in a buffer containing 1% sodium dodecyl sulfate (SDS); 20 mM Tris-HCl, pH 7.4; 5 μg/mL pepstatin A; 10 μg/mL leupeptin; 5 μg/mL aprotinin; and 1 mM phenyl-methylsulfonyl fluoride and then heated for 5 min. After passage through a 20-gauge needle ten times and centrifugation at 15,000 rpm at 4 °C for 30 min, the aliquot was boiled in a standard reducing sample buffer for 3 min and subjected to SDS-polyacrylamide gel electrophoresis. This was followed by transfer to an Immobilon-P membrane (Millipore, Burlington, MA, USA) and hybridization with an anti-DPP8 antibody (OTI1D2), monoclonal, mouse, NBP2-01830 (Novus Biologicals, Englewood, CO, USA), an anti-DPP9 antibody (OTI2E3), monoclonal, mouse, NBP2-01521 (Novus Biologicals), an anti-GSDMD antibody, monoclonal, rabbit, ab210070 (Abcam, Cambridge, UK), an anti-Cleaved Caspase-3 antibody (Asp175, 5A1E), monoclonal, rabbit, #9664S (Cell Signaling Technology, Danvers, MA, USA), an anti-DFNA5/GSDME antibody (EPR19859, N-terminal), monoclonal, rabbit, ab215191 (Abcam), an anti-Caspase-1 antibody, polyclonal, rabbit, #2225S (Cell Signaling Technology), an anti-CARD8 antibody (2108C2a), monoclonal, mouse, sc-81213 (Santa Cruz Biotechnology, Dallas, TX, USA), an anti-Caspase-3 antibody, polyclonal, rabbit, #9662S (Cell Signaling Technology), an anti-HCK antibody (E1I7F), monoclonal, rabbit, #14643 (Cell Signaling Technology), an anti-LCK antibody (3A5), monoclonal, mouse, sc-433 (Santa Cruz Biotechnology), an anti-Fyn antibody (15), monoclonal, mouse, sc-434 (Santa Cruz Biotechnology), an anti-c-Fgr antibody (B-8), monoclonal, mouse, sc-166079 (Santa Cruz Biotechnology), an anti-Lyn antibody (H-6), monoclonal, mouse, sc-7274 (Santa Cruz Biotechnology), an anti-Blk antibody (9D10D1), monoclonal, mouse, sc-65980 (Santa Cruz Biotechnology), an anti-β-actin antibody (8H10D10), monoclonal, mouse, #3700S (Cell Signaling Technology), an anti-AK2 antibody, polyclonal, rabbit, ab37594 (Abcam), an anti-FADD antibody (A66-2), monoclonal, mouse, 556402 (BD Biosciences, Franklin Lakes, NJ, USA), an anti-phospho-FADD (Ser194) antibody, polyclonal, rabbit, #2781S (Cell Signaling Technology), or an anti-DUSP26 antibody, polyclonal, rabbit, GTX109283 (GeneTex, Zeeland, MI, USA). Proteins detected by these antibodies were visualized with horseradish-peroxidase-conjugated anti-mouse or rabbit antibody (Santa Cruz Biotechnology) followed by the use of enhanced chemiluminescence (Amersham Pharmacia Biotech., Amersham, UK), as we described previously [16].

### 2.6. Microarray Analysis

RNA was isolated from cells using an RNeasy Mini Kit (QIAGEN, Hilden, Germany). Microarray analysis was performed using the 3D-Gene human oligo chip 25k (TORAY Industries, Tokyo, Japan), which permits the detection of 24,460 mRNAs. After hybridization, the DNA microarray was washed according to the manufacturer’s instructions, followed by image scanning using 3D-Gene Scanner 3000 (TORAY Industries) and data processing using 3D-Gene Extraction 2.0.0.4 (TORAY Industries).

### 2.7. In Vivo Studies

In vivo experiments were performed as we previously described [16]. NOD.Cg-Prkdc^scid^Il2rg^tm1Wjl^/SzJ (NSG) female mice of age 6–7 weeks and weight 19–21 g were obtained from Charles River Japan Inc. (Kanagawa, Japan). The mice were kept under specific pathogen-free conditions with a 12 h day and night cycle with free access to food and water, and they received humane care in compliance with Institutional Guidelines. All experiments were approved by the Animal Care and Use Committee of Toyama University (reference number A2020UH-5). In order to evaluate for lethal toxicity, NSG mice were administered with 1G244 or tominostat subcutaneously once a week, with body weights being measured at the same time. For the evaluation of antitumor activity, 5 × 10^6^ of MM.1S or Daudi cells were subcutaneously inoculated on the left side on the backs of NSG mice. Three days after the inoculation, 1G244 or tominostat was administered subcutaneously once a week. Tumor measurements were obtained at the same time with a caliper, and tumor volume was calculated according to the following formula: MD × TL^2^ × 1/2, with MD and TL being the maximum diameter and transverse length, respectively.

## 3. Results

### 3.1. DPP8-Dependent Antineoplastic Effect of High-Dose 1G244

**Figure 1:**
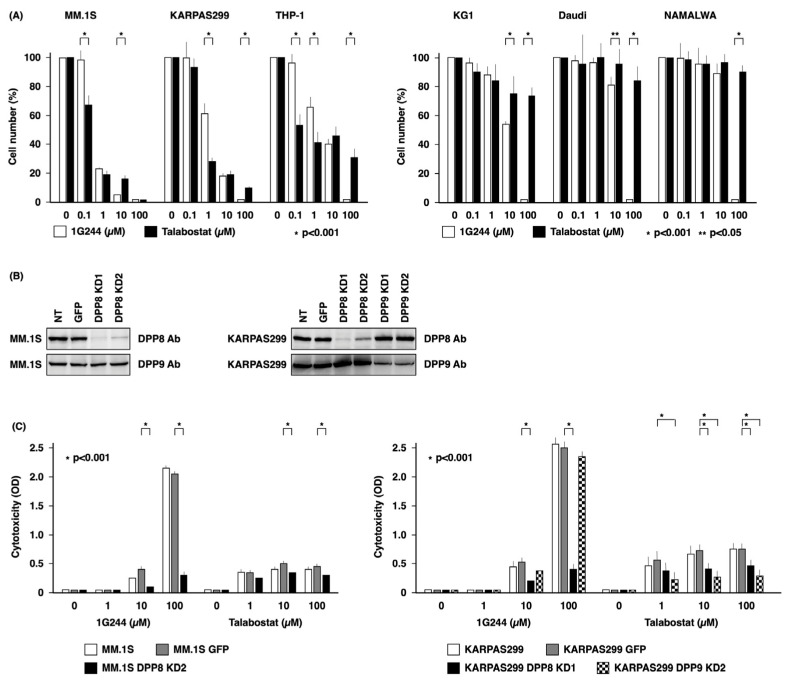
DPP8-dependent antineoplastic effect of high-dose 1G244. (**A**) 1.0 × 10^5^ of hematological cancer cell lines (MM.1S, KARPAS299, THP-1, KG1, Daudi, or NAMALWA) were cultured with DPP8/9 inhibitors (1G244 or talabostat) at doses of 0–100 µM for 72 h. Cell number was estimated by a colorimetric assay using WST-1 reagent (n = 6). (**B**) Knockdown studies of DPP8 and DPP9 in MM.1S or KARPAS299 cells. NT, no treatment; GFP, control vector; KD, knockdown. Expression level of DPP8 or DPP9 was estimated by Western blot analysis. (**C**) 1.0 × 10^5^ of MM.1S and KARPAS299 cells and their transfectants were cultured with DPP8/9 inhibitors (1G244 or talabostat) at doses of 0–100 µM for 6 h. Cytotoxicity was estimated by a LDH release assay (n = 6).

We compared the cytotoxic effect of the DPP8/9 inhibitors 1G244 and talabostat on hematological cancer cell lines using water-soluble tetrazolium (WST) to detect the respiratory chain metabolic activity in viable cells. As shown in Figure 1A, in three cell lines sensitive to DPP8/9 inhibitors, specifically MM.1S, KARPAS299, and THP-1 cells, talabostat was superior to 1G244 at low concentrations of 0.1 and 1 µM. On the other hand, the opposite was true at concentrations as high as 10 and 100 µM, with 1G244 being more effective than talabostat. Meanwhile, talabostat exhibited a minimal cytotoxic effect against the three less sensitive cell lines, specifically KG1, Daudi, and NAMALWA cells. However, 1G244 was effective at concentrations of 10 and 100 µM against these cell lines.

To determine whether the DPP8/9 inhibitors 1G244 and talabostat exerted their cytotoxic effect through DPP8 or DPP9, we conducted knockdown studies on DPP8 or DPP9 expression in MM.1S and KARPAS299 cells, which are highly sensitive cell lines. As shown in Figure 1B, DPP8 was effectively suppressed in MM.1S cells. On the other hand, MM.1S cells with DPP9 knockdown did not proliferate and died. DPP8 and DPP9 in KARPAS299 cells were both effectively suppressed.

As shown in Figure 1C, the cytotoxic effect of DPP8/9 inhibitors was assessed by measuring lactate dehydrogenase (LDH) released from dead cells into the culture supernatant. In both MM.1S GFP and KARPAS299 GFP cells transfected with the control vector, talabostat at a low concentration of 1 µM and 1G244 at a high concentration of 100 µM exhibited greater cytotoxic effects than the others at the same concentrations, consistent with results shown in Figure 1A. On the other hand, both KARPAS299 DPP8 and DPP9 knockdown cells were less sensitive to 10 and 100 µM of talabostat, which was particularly more pronounced in DPP9 knockdown cells. MM.1S DPP8 knockdown cells also showed decreased sensitivity to talabostat. The selective reduced sensitivity of KARPAS299 DPP8 knockdown cells to 100 µM of 1G244 was also demonstrated. Greatly reduced sensitivity was also observed in MM.1S DPP8 knockdown cells. This DPP8-knockdown-induced decrease in sensitivity to 1G244 was also observed in KARPAS299 and in MM.1S at 10 µM. These results showed that the cytotoxic effect of talabostat depends mainly on DPP9, as well as partly on DPP8, and that the potent anticancer activity of 1G244 at high concentrations is dependent on DPP8.

### 3.2. Caspase-3-Mediated Apoptosis as Anticancer Effect by High-Dose 1G244

**Figure 2:**
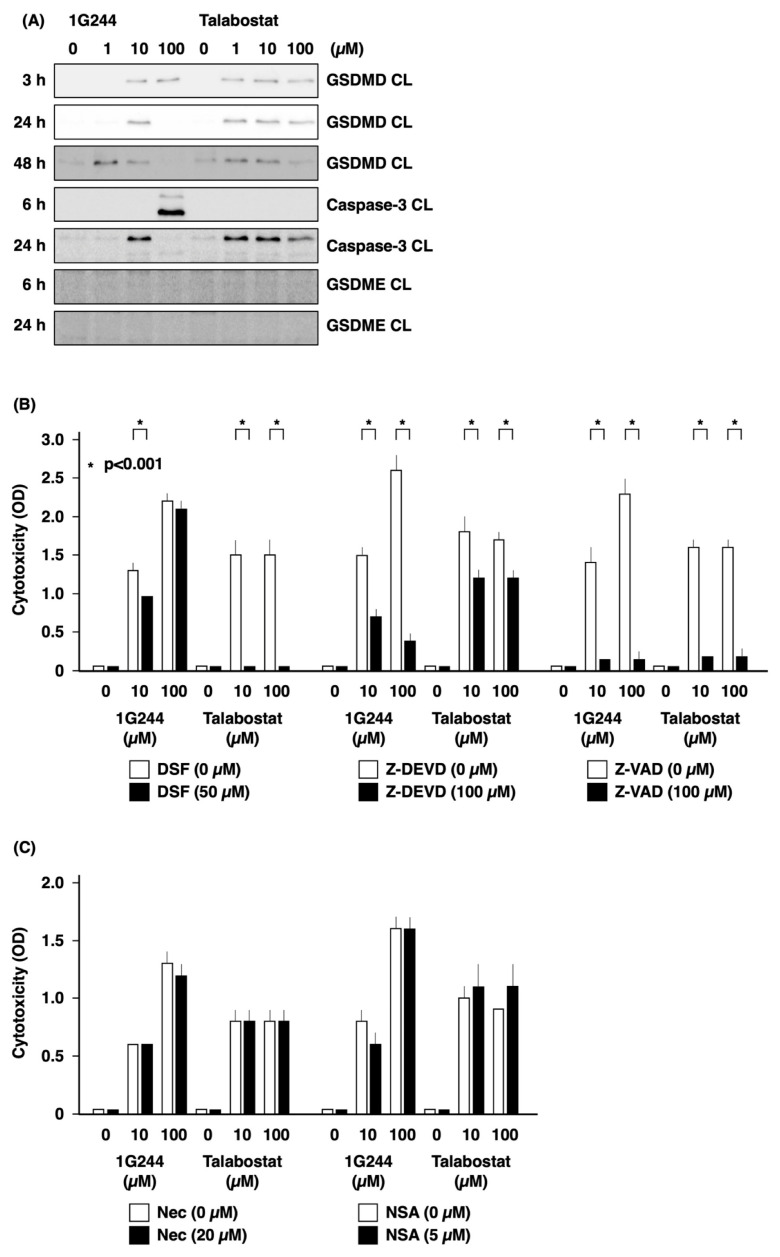
Caspase-3-mediated apoptosis as anticancer effect by high-dose 1G244. (**A**) 1 × 10^6^ MM.1S cells were cultured with DPP8/9 inhibitors (1G244 or talabostat) at doses of 0–100 µM for 3–48 h. The cleaved form (CL) of gasdermin-D (GSDMD), caspase-3, or gasdermin-E (GSDME) was detected by Western blot analysis. The upper caspase-3 CL: 19 kDa; and the lower caspase-3 CL: 17 kDa. (**B**,**C**) 1.0 × 10^5^ of MM.1S cells were cultured with DPP8/9 inhibitors (1G244 or talabostat) at doses of 0–100 µM for 6 h with disulfiram (DSF), Z-DEVD, Z-VAD, necrostatin-1 (Nec), or necrosulfonamide (NSA). Cytotoxicity was estimated by a LDH release assay (n = 6).

Figure 2A shows the results of our investigation into the types of cell death caused by DPP8/9 inhibitors. In MM.1S cells, cleaved GSDMD, a marker of pyroptosis, was detected between 3 and 48 h of treatment by 1 to 100 µM talabostat. Cleaved GSDMD was also detected following 1G244 exposure. On the other hand, cleaved caspase-3, a marker of apoptosis, was detected following 24 h of exposure to 10 µM 1G244, while a greater level was detected at an earlier time point of 6 h after stimulation with 100 µM. Cleaved caspase-3 was also detected following 24 h of exposure to 1 to 100 µM of talabostat. These results suggested that DPP8/9 inhibitors induce the cleavage of GSDMD, as well as caspase-3, and that the potent cytotoxic effect of high concentrations of 1G244 may be dependent on caspase-3.

Figure 2B shows the findings from experiments conducted to examine these hypotheses. Disulfiram (DSF) almost completely inhibited cell death induced by 10 and 100 µM of talabostat, while weakly suppressing 1G244 at 10 µM. Cell death suppressed by DSF is pyroptosis mediated by GSDMD pore formation. In contrast, 1G244 at concentrations of 10 and 100 µM was largely unaffected by DSF but was strongly inhibited by the caspase-3/7 inhibitor Z-DEVD.

These results were consistent with our hypothesis that the strong anticancer effect exhibited by high concentrations of 1G244 is caspase-3-dependent apoptosis. At the same time, Z-DEVD also weakly inhibited 10 and 100 µM of talabostat, suggesting that caspase-3-mediated pyroptotic pathways may also be involved. While a potential candidate may involve the cleavage of Gasdermin-E (GSDME) [18], our work did not implicate this pathway (Figure 2A). Of note is the fact that talabostat at 10 and 100 µM was strongly inhibited by the pan caspase inhibitor Z-VAD. This observation was consistent with the fact that caspase-1 is required for pyroptosis in the canonical inflammasome pathway and caspase-4/5/11 in the noncanonical inflammasome pathway [18]. Meanwhile, no inhibitory effect was observed for necrostatin-1 (Nec) or necrosulfonamide (NSA), inhibitors of necroptosis (Figure 2C).

### 3.3. Dependence on HCK for DPP8/9 Inhibitor-Induced Pyroptosis

**Figure 3:**
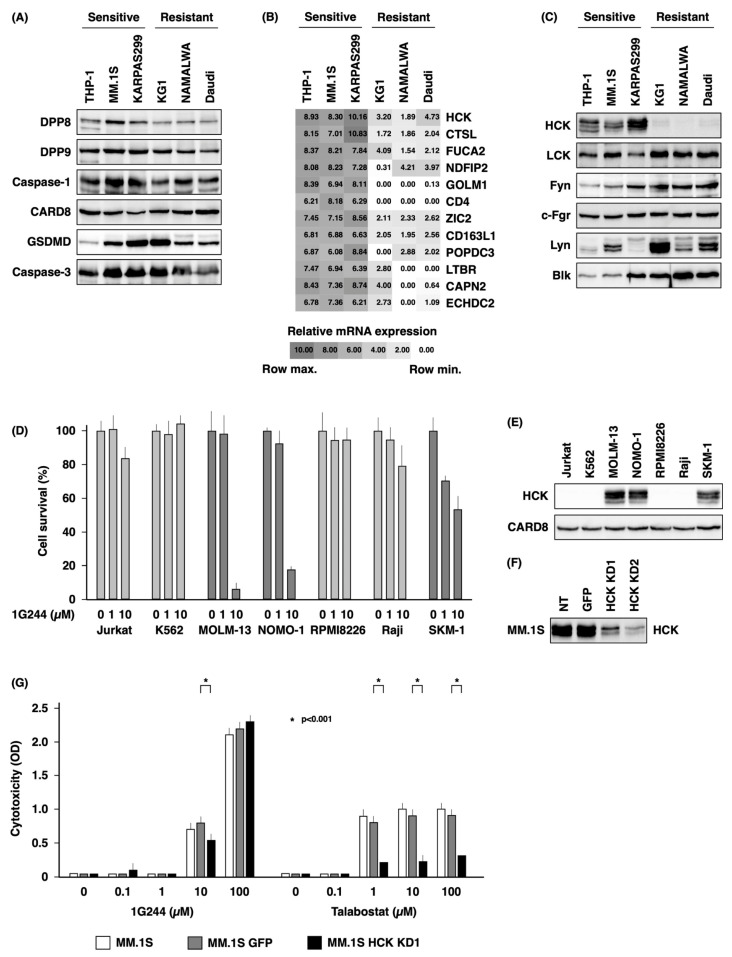
Dependence on HCK for DPP8/9 inhibitor-induced pyroptosis. (**A**) Expression level of DPP8, DPP9, caspase-1, CARD8, GSDMD, or caspase-3 in THP-1, MM.1S, KARPAS299, KG1, NAMALWA, or Daudi cells was estimated by Western blot analysis. (**B**) Gene expression of THP-1, MM.1S, KARPAS299, KG1, NAMALWA, or Daudi cells was analyzed by microarray method using 3D-Gene. (**C**) Expression level of HCK, LCK, Fyn, c-Fgr, Lyn, or Blk in THP-1, MM.1S, KARPAS299, KG1, NAMALWA, or Daudi cells was estimated by Western blot analysis. (**D**) 1.0 × 10^5^ of hematological cancer cell lines (Jurkat, K562, MOLM-13, NOMO-1, RPMI8226, Raji, or SKM-1) were cultured with 1G244 at doses of 0–10 µM for 72 h. Cell number was estimated by a colorimetric assay using WST-1 reagent (n = 6). (**E**) Expression level of HCK or CARD8 in Jurkat, K562, MOLM-13, NOMO-1, RPMI8226, Raji, or SKM-1 cells was estimated by Western blot analysis. (**F**) Knockdown studies of HCK in MM.1S cells. NT, no treatment; GFP, control vector; KD, knockdown. Expression level of HCK was estimated by Western blot analysis. (**G**) 1.0 × 10^5^ of MM.1S cells and their transfectants were cultured with DPP8/9 inhibitors (1G244 or talabostat) at doses of 0–100 µM for 6 h. Cytotoxicity was estimated by a LDH release assay (n = 6).

Figure 3A shows the results of experiments evaluating possible predictors of sensitivity to DPP8/9 inhibitors. The sensitive cell lines studied were THP-1, MM.1S, and KARPAS299, and resistant cell lines studied were KG1, NAMALWA, and Daudi. We first examined the expression of DPP8 and DPP9 as prerequisite factors and caspase-1, CARD8, and GSDMD as factors related to pyroptosis signaling, as well as caspase-3 as a factor involved in apoptosis signaling. However, no association was found between the expression level of any of the factors and susceptibility.

Microarray analysis was then performed as a comprehensive evaluation to compare sensitive cell lines with resistant cell lines. Factors with high expression in the former and low expression in the latter are shown in Figure 3B. Among these, we paid particular attention to hematopoietic cell kinase (HCK). HCK is one of the Src-family tyrosine kinases and mediates proliferation, survival, and adhesion signals sent by cell surface receptors [19]. Furthermore, HCK has been reported to mediate caspase-3-mediated apoptosis [20]. We therefore decided to further investigate the possible involvement of HCK.

Our initial studies employed the Western blotting method to examine the HCK protein level in each cell line, as shown in Figure 3C. HCK was selectively highly expressed in the three highly sensitive cell lines. In contrast, there was no association with the susceptibility and expression of other members of the Src-family tyrosine kinases with known expression in hematopoietic cells, including LCK, Fyn, c-Fgr, Lyn, and Blk (Figure 3C).

Meanwhile, MOLM-13, NOMO-1, and SKM-1 were sensitive to 1G244, while Jurkat, K562, RPMI8226, and Raji were resistant to 1G244 (Figure 3D), with HCK being highly expressed in a selective manner in the three sensitive cell lines (Figure 3E). On the other hand, CARD8, which has been reported to define DPP8/9 inhibitor sensitivity [13], was almost equally expressed in these seven cell lines.

To further confirm the dependence on HCK for DPP8/9 inhibitor sensitivity, we conducted HCK knockdown studies in highly sensitive MM.1S cells (Figure 3F). The knockdown cells showed reduced sensitivity to 10 µM of 1G244 and to 1, 10, and 100 µM of talabostat. However, no effect on sensitivity to 100 µM of 1G244 was observed (Figure 3G). These results indicated that DPP8/9-mediated pyroptosis is HCK-dependent, but HCK is not involved in DPP8-mediated apoptosis induced by high levels of 1G244. In addition, the forced expression of HCK in hyposensitive Daudi and NAMALWA (Figure S1A) did not alter their sensitivity to 1G244 or talabostat. (Figure S1B,C). These results suggested that the presence of HCK is a necessary but not sufficient condition for DPP8/9-inhibitor-induced pyroptosis.

### 3.4. Antitumor Effects of Tominostat

High concentrations of 1G244 induced apoptotic cell death via DPP8, even in blood cancer cell lines resistant to pyroptosis induced by talabostat and low concentrations of 1G244 via DPP8/9. We therefore attempted to determine whether increasing the DPP8 selectivity of 1G244 could result in a greater antineoplastic effect. The selectivity for DPP8 compared to DPP9 is expressed as the selectivity index (SI), i.e., IC50 for DPP9 divided by IC50 for DPP8. The SI of 1G244 is 3.8 [21], whereas the SI of 12 m with the H in the piperazine skeleton of 1G244 replaced by (S)CH3 is reported to be 8.1 [17]. We named this 12 m molecule tominostat and performed the experiments described below.

**Figure 4:**
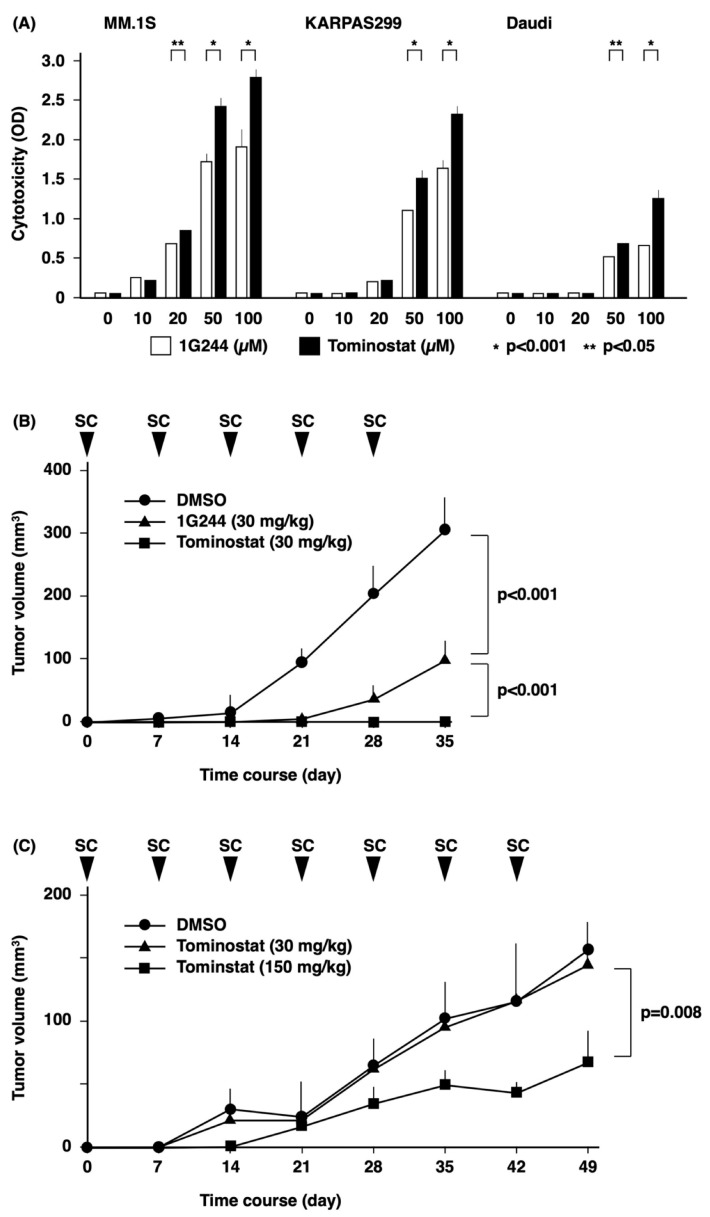
Antitumor effects of tominostat. (**A**) 1.0 × 10^5^ of MM.1S, KARPAS299, or Daudi cells were cultured with DPP8/9 inhibitors (1G244 or tominostat) at doses of 0–100 µM for 6 h. Cytotoxicity was estimated by a LDH release assay (n = 6). (**B**,**C**) 5 × 10^6^ of MM.1S (**B**) or Daudi (**C**) cells were subcutaneously inoculated into NSG mice (n = 6). Three days after inoculation, 1G244 or tominostat was administered subcutaneously once a week. Tumor volume was assessed at the same time.

The antitumor effect of tominostat was compared to that of 1G244 in the sensitive cell lines MM.1S and KARPAS299 and in the resistant cell line Daudi in in vitro studies (Figure 4A). For all of these cell lines, at concentrations >10 µM, tominostat exhibited a greater antitumor effect as compared to the original 1G244. These results indicated that the antitumor effect at high concentrations, which is a characteristic of 1G244, is accentuated by increasing DPP8 selectivity.

**Figure 5:**
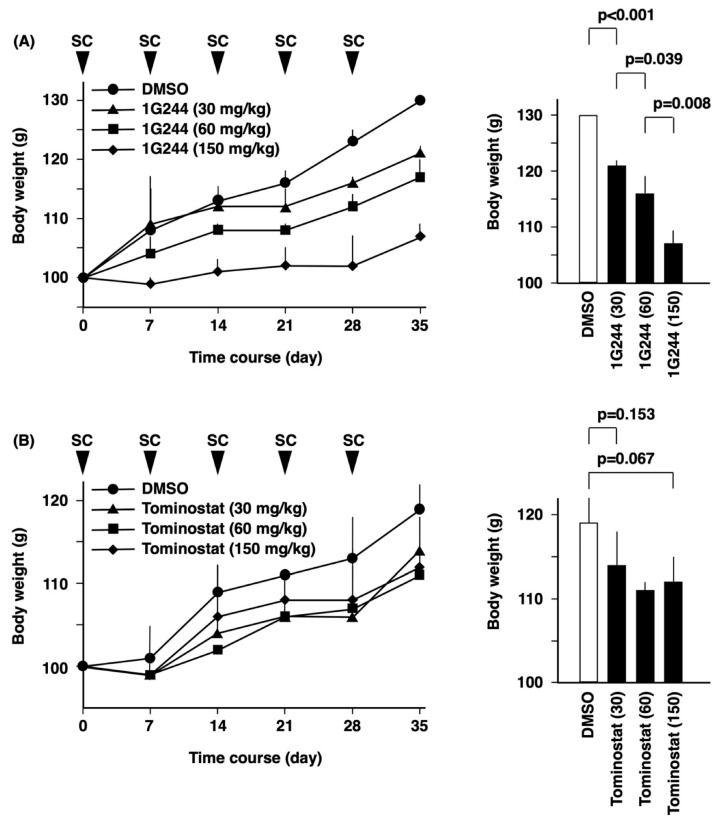
Weight loss as a side effect of tominostat treatment. NSG mice were treated with 1G244 (**A**) or tominostat (**B**) subcutaneously once a week (n = 6). The body weight was assessed at the same time.

For in vivo studies, we first conducted experiments examining the toxicity profile of the pertinent inhibitors. There was no death of the tested animals from acute toxicity from the administration of either 1G244 (Figure 5A) or tominostat (Figure 5B). However, dose-dependent weight loss was observed with 1G244. On the other hand, this weight-loss toxicity was not observed with tominostat, even when the dose was increased to 150 mg/kg.

The next in vivo experiment examined the antitumor effect of the inhibitors. Although the treatment of mice implanted subcutaneously with the sensitive cell line MM.1S cells with 30 mg/kg of 1G244 or tominostat resulted in tumor growth inhibition, tominostat exhibited significantly superior antitumor activity (Figure 4B). Meanwhile, the treatment of mice transplanted with the resistant cell line Daudi with 30 mg/kg tominostat did not inhibit tumor growth. However, an increase in treatment dose to 150 mg/kg tominostat led to significant Daudi cell tumor shrinkage (Figure 4C). These results showed that tominostat has superior antineoplastic activity as compared to the original 1G244 and that it exhibits effectiveness against resistant cells at higher doses without the side effect of weight loss.

## 4. Discussion

An initial finding of our current work is the dual nature of 1G244. At low concentrations, 1G244 induces pyroptosis mainly through the inhibition of DPP9, while it induces apoptosis through the inhibition of DPP8 at high concentrations. A potential explanation for this duality is that 1G244 inhibits DPP8 and DPP9 by different mechanisms. Indeed, it has been suggested that 1G244’s inhibition of DPP8 is based on binding that is slow and tight [21], i.e., irreversible [11], while its inhibition of DPP9 is competitive [21]. However, studies using experimental 3D models have not demonstrated clear differences between the molecular structures of 1G244-liganded DPP8 and 1G244-liganded DPP9 to support the suggested hypothesis [22]. Such differences may become apparent in studies using tominostat, an analog of 1G244 with increased selectivity for DPP8. We are currently planning to analyze the molecular structures of tominostat-liganded DPP8/9 to further evaluate this important issue.

The dependence of cell death caused by high concentrations of 1G244 on DPP8 inhibition is precisely demonstrated by DPP8 knockdown experiments (Figure 1C). Further evidence supporting this conclusion is based on the enhanced cytotoxic effect of high concentrations of DPP8-selective tominostat (Figure 4A). Whether this enhanced cytotoxicity can lead to use in clinical practice as an anticancer agent depends largely on the toxicity profile and severity of adverse events. Toxicity of 1G244 has been reported for daily intravenous administration for 14 days to rats [21]. Groups administered with doses of 1 and 10 mg/kg showed hematological and serum parameters that were all within normal limits. However, 30 mg/kg dosing led to treatment discontinuation from such dose-limiting toxicities as startle reflexes, chronic and tonic convulsions, and opisthonos.

In our previous and present studies, no such toxicity was observed with 1G244 treatment, likely due to the fact it was administered subcutaneously once a week in mice [16]. However, clear weight loss was observed in the 30 mg/kg group, which worsened with increasing doses of 60 mg/kg and 150 mg/kg (Figure 5). In contrast, no weight loss was observed with tominostat in any of the 30, 60, or 150 mg/kg groups.

A reason for the observed reduction in toxicity may be due to differences in the tissue expression of DPP8 and DPP9. Specifically, DPP9 is expressed throughout all organs in the body (Dataset: GeneAtlas U133A, gcrma), with its absence in mice causing impairment in tongue development, suckling defects, and subsequent neonatal lethality [23,24]. On the other hand, DPP8 expression is relatively restricted to hematopoietic cells (Dataset: GeneAtlas U133A, gcrma).

Importantly, the low-sensitivity cell line Daudi was resistant to 100 µM of 1G244, but the same dose of tominostat exhibited effective in vitro antitumor activity (Figure 4A), and 150 mg/kg tominostat inhibited tumor growth in vivo (Figure 4C). These findings indicate that the increased DPP8 selectivity of tominostat compared to 1G244 not only enhanced its antitumor effect, but also reduced its toxicity, allowing higher doses to be safely administered to treat less sensitive tumor cells.

An issue that needs to be clarified involves the specific signaling events induced by DPP8 inhibition. Comparing high- and low-susceptible cell lines, we identified HCK as one factor defining differences in susceptibility. However, HCK knockdown suppressed pyroptosis induced by DPP9 inhibition, while apoptosis induced by DPP8 inhibition was unaffected (Figure 3).

Recent work by others suggested a mechanism for HCK involvement in signaling events of pyroptosis [25]. These authors demonstrate that the genetic and pharmacological inhibition of HCK suppresses NLRP3 inflammasome activation and that HCK binds to the NBD and LRR domains of NLRP3. In addition, DPP9 is shown to regulate pyroptosis by binding to Nlrp1b [15]. Our current findings that HCK dictates susceptibility to pyroptosis induced by DPP9 inhibition are therefore consistent with previously published reports.

On the other hand, assuming that DPP8 inhibition induces apoptosis by inhibiting the cleavage of relevant substrates, adenylate kinase 2 (AK2), which is involved in energy metabolism, can be considered as a potential candidate substrate. AK2 forms a complex with dual-specificity phosphatase 26 (DUSP26) to dephosphorylate fas-associated protein with death domain (FADD) [26]. Phosphorylated FADD plays an essential role in cellular mechanisms that enhance chemotherapy-induced apoptosis [27]. AK2 is one of the representative substrates of DPP8 [1,28]. It is possible that the inhibition of AK2 cleavage by DPP8 leads to increased levels of phosphorylated FADD.

Our preliminary examination showed that AK2, FADD, phosphorylated FADD, and DUSP26 were all equally expressed in the three high-sensitive and three low-sensitive cell lines used in Figure 1A (Figure S2). These findings are consistent with results showing that all six of these cell lines were equally sensitive to high concentrations of 1G244 (Figure 1A). We are currently planning future in vitro reconstitution experiments involving these factors.

## 5. Conclusions

**Figure 6:**
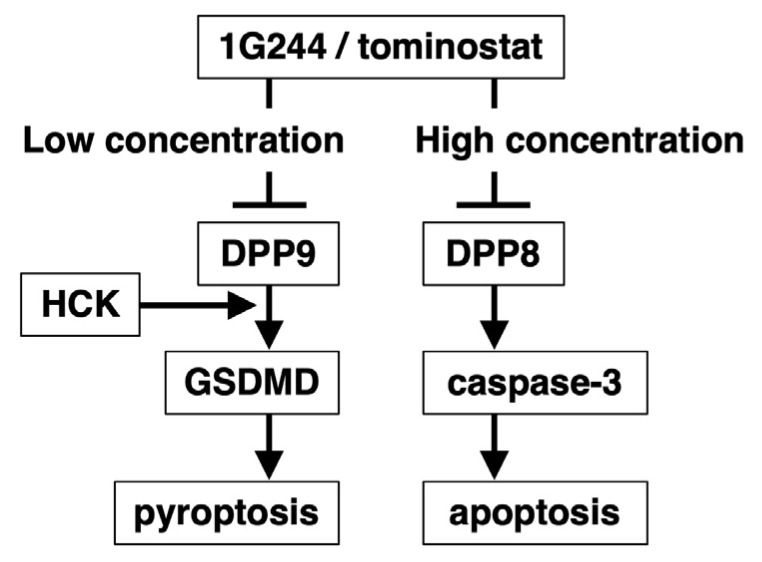
A diagram describing the dual signaling nature of 1G244/tominostat.

In this paper, we show that the DPP8/9 inhibitor 1G244 exhibits a dual role: at low concentrations, it induces DPP9 inhibition-dependent pyroptosis, and at high concentrations, it induces DPP8 inhibition-dependent apoptosis. Importantly, we identified HCK expression as a necessary determinant of susceptibility to pyroptosis, but with no effect on the induction of apoptosis (Figure 6). Furthermore, a greater antitumor effect associated with the lower toxicity profile of tominostat, a methylpiperazine analog of 1G244 with high DPP8 selectivity, was observed and compared to 1G244. Our findings suggest that additional enhancement in DPP8 selectivity by further modifications of tominostat chemical structure may lead to the development of novel anticancer agents that are effective against a wide range of hematologic malignancies.

## Data Availability

Data are available on reasonable request. All data relevant to the study are included in the article or uploaded as online Supplementary Materials.

## Supplementary Materials

The following supporting information can be downloaded at: https://www.mdpi.com/article/10.3390/cells12071100/s1, Figure S1: Effect of forced expression of HCK in resistant cell lines on DPP8/9 inhibitor-induced cytotoxicity, Figure S2: Expression of AK2-mediated apoptosis-inducing factors.
