# Supplementary material for: DPP8 Selective Inhibitor Tominostat as a Novel and Broad-Spectrum Anticancer Agent against Hematological Malignancies
Source: Cells. 2023 Apr 6;12(7):1100. doi: 10.3390/cells12071100 (PMC10093441; doi:10.3390/cells12071100)
Supplement: Supplementary file 1 [file cells-12-01100-s001.zip › cells-2293524-supplementary.pdf]

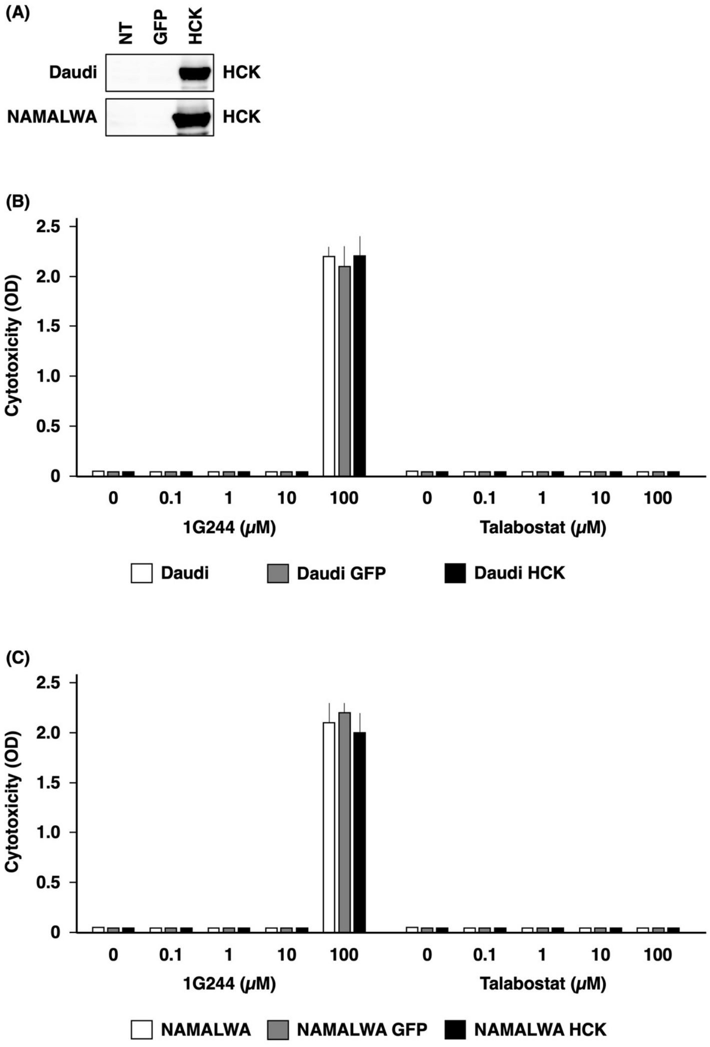

Figure S1: Effect of forced expression of HCK in resistant cell lines on DPP8/9 inhibitor-induced cytotoxicity. (A) The forced overexpression of HCK in Daudi and NAMALWA cells was estimated by Western blot analysis. NT, no treatment; GFP, control vector. (B and C) 1.0×10^5 of Daudi or NAMALWA cells and their transfectants were cultured with DPP8/9 inhibitors (1G244 or talabostat) at doses of 0-100 μM for 6 hours. Cytotoxicity was estimated by a LDH release assay (n=6).

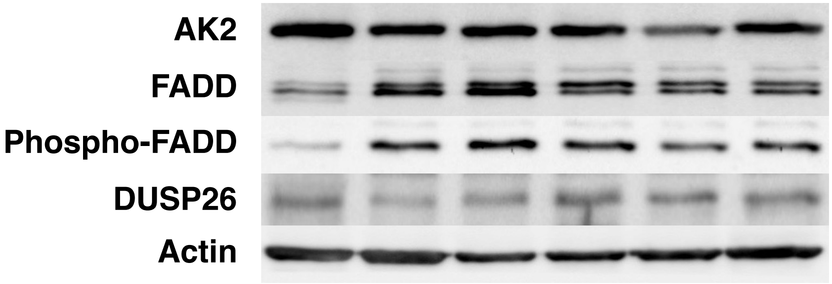

Figure S2: Expression of AK2-mediated apoptosis-inducing factors. Expression level of AK2, FADD, phosphor-FADD, and DUSP26 in THP-1, MM.1S, KARPAS299, KG1, NAMALWA, or Daudi cells was estimated by Western blot analysis.
